# Host Alternation Is Necessary to Maintain the Genome Stability of Rift Valley Fever Virus

**DOI:** 10.1371/journal.pntd.0001156

**Published:** 2011-05-24

**Authors:** Sara Moutailler, Benjamin Roche, Jean-Michel Thiberge, Valérie Caro, François Rougeon, Anna-Bella Failloux

**Affiliations:** 1 Molecular Genetics of Bunyavirus, Department of Virology, Institut Pasteur, Paris, France; 2 Developmental Genetics and Biochemistry, Department of Immunology, Institut Pasteur, Paris, France; 3 Genotyping of Pathogens and Public Health, Department of Infection and Epidemiology, Institut Pasteur, Paris, France; Texas Biomedical Research Institute, United States of America

## Abstract

**Background:**

Most arthropod-borne viruses (arboviruses) are RNA viruses, which are maintained in nature by replication cycles that alternate between arthropod and vertebrate hosts. Arboviruses appear to experience lower rates of evolution than RNA viruses that replicate in a single host. This genetic stability is assumed to result from a fitness trade-off imposed by host alternation, which constrains arbovirus genome evolution. To test this hypothesis, we used Rift Valley fever virus (RVFV), an arbovirus that can be transmitted either directly (between vertebrates during the manipulation of infected tissues, and between mosquitoes by vertical transmission) or indirectly (from one vertebrate to another by mosquito-borne transmission).

**Methodology/Principal Findings:**

RVFV was serially passaged in BHK21 (hamster) or Aag2 (*Aedes aegypti*) cells, or in alternation between the two cell types. After 30 passages, these single host-passaged viruses lost their virulence and induced protective effects against a challenge with a virulent virus. Large deletions in the *NSs* gene that encodes the virulence factor were detectable from the 15^th^ serial passage onwards in BHK21 cells and from the 10^th^ passage in Aag2 cells. The phosphoprotein *NSs* is not essential to viral replication allowing clones carrying deletions in *NSs* to predominate as they replicate slightly more rapidly. No genetic changes were found in viruses that were passaged alternately between arthropod and vertebrate cells. Furthermore, alternating passaged viruses presenting complete *NSs* gene remained virulent after 30 passages.

**Conclusions/Significance:**

Our results strongly support the view that alternating replication is necessary to maintain the virulence factor carried by the *NSs* phosphoprotein.

## Author Summary

Arthropod-borne viruses are transmitted among vertebrate hosts by insect vectors. Unusually, Rift Valley fever virus (RVFV) can also be transmitted by direct contacts of animals/humans with infectious tissues. What are the molecular mechanisms and evolutionary events leading to adopt one mode of transmission rather than the other? Viral replication is implied to be different in a vertebrate host and an invertebrate host. The alternating host cycle tends to limit virus evolution by adopting a compromise fitness level for replication in both hosts. To test this hypothesis, we used a cell culture model system to study the evolution of RVFV. We found that freeing RVFV from alternating replication in mammalian and mosquito cells led to large deletions in the *NSs* gene carrying the virulence factor. Resulting *NSs*-truncated viruses were able to protect mice from a challenge with a virulent RVFV. Thus, in nature, virulence is likely maintained by continuous alternating passages between vertebrates and insects. Thereby, depending on the mode of transmission adopted, the evolution of RVFV will be of major importance to predict the outcome of outbreaks.

## Introduction

Most arthropod-borne viruses (arboviruses) are RNA viruses, although they use a variety of strategies to ensure their replication and transmission. The feature that best distinguishes RNA genomes from DNA ones is the high mutation rate of the former during replication. Misincorporation errors during replication have been estimated to occur within the range of 10^−3^–10^−5^ substitutions per nucleotide and per round of copying [1]. The main factor contributing to such high mutation rates is a lack of proof-reading repair activities that is associated with RNA replicases [2]. Another source of mutations results from the spontaneous deamination of Cytidine residues to Uracil. In DNA genomes, this reaction is repaired by a Uracil-glycosylase, but this cannot function on an RNA template [3]. The resulting complex mixtures of closely related RNA genomes are termed quasispecies [4], [5] whose existence allow RNA viruses to adapt rapidly to fluctuating environments [6], [7]. Indeed, mutation rates per nucleotide site of around 10^−4^ mean that for a 10 kb genome, an average of one mutation is incorporated each time the genome is copied, and it is this, together with short replication times and large population sizes, that ensure the existence of the quasispecies genome pool.

However, sequence comparisons reveal that RNA arboviruses are relatively stable in nature, suggesting that the alternating host cycle (between vertebrate and invertebrate hosts) constrains viral evolution by a strong conservative sequence selection. This sequence stability may result from the requirements for replication in two separate hosts that present conflicting niches for replication and adaptation [8]. Furthermore, low rates of evolution do not necessarily reflect the adaptive compromise of a virus to the alternating host cycle [9], but could be principally related to other biological constraints including the need to maintain virulence [10], [11]. Effectively, more virulent viral strains are generally at a competitive advantage in mixed-strain infections [12]. Virulence can be considered as a consequence of virus efforts to maximize its fitness: the virus must replicate extensively in a host to ensure its transmission to the next host. However, viral replication damages host tissues, leading to host death, which can be considered as seriously deleterious for virus survival [13], [14]. Alternation may play a significant role in maintaining the genetic stability of arboviruses; setting aside one or several selective filters may lead to accelerate evolution. For such, we used a cell culture system for these studies, as *in vitro* systems are convenient to investigate the evolution of arboviruses.

Contrary to most arboviruses, Rift Valley fever virus (RVFV), a member of the *Phlebovirus* genus within the *Bunyaviridae* family can be transmitted through direct contact with body fluids or aborted fetuses. It constitutes an interesting model to study host alternating cycling as cause of genetic stability of arboviruses in nature. RVFV is a tri-segmented negative-stranded RNA virus composed of the L segment that codes for the RNA-dependent RNA polymerase, the M segment that codes for the *G_N_* and *G_C_* glycoprotein precursor, and the S segment that has an ambisense strategy, coding for the *N* nucleoprotein and the *NSs* phosphoprotein [15]. The *NSs* phosphoprotein plays a key role in RVFV pathogenesis in the mammalian host. A natural isolate defective for the *NSs* protein (Clone 13; [16]), was found to be avirulent for mice [17] and to induce an interferon response in mammalian cells, in contrast to virulent RVFV strains [18].

Here, we present results showing that genetic material non-essential to viral replication such as the phosphoprotein *NSs* is rapidly eliminated leading to the loss of virulence.

## Materials and Methods

### Ethics Statement

The Institut Pasteur animal facility has received accreditation from the French Ministry of Agriculture to perform experiments on live mice in appliance of the French and European regulations on care and protection of the Laboratory Animals. This study was approved by the relative IACUC at the Institut Pasteur.

### Cell cultures

We used two cell lines: a mammalian cell line derived from hamster kidney (BHK21) and an insect cell line derived from *Aedes aegypti* larvae (Aag2). BHK21 cells defective in IFN-a/b signaling were grown at 37°C with 5% CO_2_ in Glasgow's minimal essential medium (G-MEM) containing 5% fetal bovine serum (FBS), 1000 units/mL penicillin, 1 mg/mL streptomycin, tryptose phosphate broth 1× and HEPES 0.01M. Monolayer cultures of Aag2 cells were grown at 28°C in Schneider Drosophila medium supplemented with 10% heat-inactivated FBS, L-Glutamine 0.4×, 1000 units/mL penicillin and 1 mg/mL streptomycin.

### Virus strains

The parental P strain was derived from the ZH548 strain originally isolated in 1977 from a human case in Egypt [19] and passaged three times in Vero cells. The virulence of this strain is related to the phosphoprotein *NSs*, which is responsible for a general inhibition of cellular RNA synthesis by interacting with the p44 subunit of the TFIIH transcription factor [20]. Furthermore, *NSs* strongly antagonizes IFN-ß production [17], [18], [21], [22]. The titer of the frozen virus stock was 10^6.8^ PFU (plaque forming unit)/mL. Moreover, the other virus strains were produced at high titers: Z30Alt at 10^8.3^ PFU/mL, Z30B at 10^7.6^ PFU/mL and Z30A at 10^8.5^ PFU/mL. Viral titers were estimated by serial 10-fold dilutions on Vero cells.

In addition, biological clones Z30AC and Z30BC were produced by plaque purification and amplification in Vero cells from the 30^th^ serial passage of parental P strain in Aag2 cells (Z30A strain) and BHK21 cells (Z30B strain), respectively. Briefly, six-well plates containing confluent monolayers of Vero cells were infected with serial 10-fold dilutions of virus. Cells were incubated for five days under an overlay consisting of Dulbecco's MEM (DMEM), 2% FBS, antibiotics and 1% agarose at 37°C. The lytic plaques were localized and removed by suction using a pipette. Each agarose plug that contained an individual clone was dissolved overnight at +4°C in DMEM supplemented with 10% FBS before being re-amplified in BHK21 or Aag2 cells, respectively [23]. Both clones were produced at high titers: 10^8.8^ PFU/mL for Z30BC and 10^8.7^ PFU/mL for Z30AC.

### Serial/alternating passaging of virus

The parental P strain virus was subjected to 30 serial passages in BHK21 cells or Aag2 cells, or 30 passages that alternated between BHK21 and Aag2 cells (i.e. 15 passages in BHK21 cells and 15 passages in Aag2 cells), at a multiplicity of infection (MOI) of 0.1 PFU/cell. Virus was adsorbed for 1 hr onto confluent cell monolayers prepared in plastic flasks of 25 cm^2^, at 28°C for Aag2 and at 37°C for BHK21. After adsorption, the inoculum was removed, cells were washed with medium, 13 mL of maintenance medium (with 2% FBS) was added and cells were incubated at the appropriate temperature. Cell supernatants were harvested when titers reached a plateau (Figure S1): at 48 hr p.i. for BHK21 and 96 hr p.i. for Aag2 cells. At each passage, supernatants were harvested and stored in aliquots at −80°C for titration on Vero cells.

### Viral replication curve analysis

Plastic flasks of 75 cm^2^ containing confluent cell monolayers (Aag2 or BHK21) were infected at a MOI of 0.1 PFU/cell as described above. The supernatant from a flask was harvested every 2 hr from 0 to 12 hr p.i. and every 24 hr from 0 to 120 hr p.i., and titrated on Vero cells by serial 10-fold dilution [24].

### RT-PCR amplification and sequencing

Total RNA was extracted from aliquots of supernatants (100 µL) using the Nucleospin RNA II kit (Macherey-Nagel) according to the manufacturer's instructions, and RT-PCR targeting the *NSs* gene was conducted using the Titan One Tube RT-PCR kit (Roche Applied Science) following the manufacturer's recommendations. Primers were selected in the *NSs* gene that lies within the S genome segment. The amplification program was performed as follows: reverse transcription at 50°C for 30 min, an inactivation of RT enzyme step at 95°C for 3 min, followed by 35 cycles of 95°C 30 s, 51°C 30 s, 72°C 1 min, and a final step at 72°C for 5 min. The size of the PCR product was 781 bp. PCR products were excised from the gel and eluted using the QIAquick Gel Extraction Kit (Qiagen) as specified by the manufacturer. The recovered DNA was cloned into the Topo TA vector and transformed into Top10 competent cells according to the manufacturer's protocol. Colonies were screened by direct PCR, using insert-specific primers. Plasmid DNA was purified using a QIAprep Spin Miniprep kit (Qiagen), as specified by the manufacturer. Sequencing was carried out using virus-specific primers. In addition, the three segments (S, M and L) of the parental P strain and the selected strains (Z30Alt, Z30B and Z30A) were completely sequenced. For each segment, primers were designed (based on the nucleotide sequence of the reference strain ZH548) in order to obtain around 700 pb RT-PCR amplicons with an overlap of around 100 pb along the entire segment (Table S1). Amplicons were obtained using SuperScript One-Step RT-PCR with platinium Taq (Invitrogen) following the manufacturer's recommendations. The amplification program was performed as follows: reverse transcription at 50°C for 30 min, an inactivation of RT enzyme step at 94°C for 2 min, followed by 35 cycles of 94°C 15 s, 50°C 30 s, 72°C 1 min 30, and a final step at 72°C for 10 min. The obtained fragments were purified by ultrafiltration (Millipore). Sequencing reactions were performed using the BigDye Terminator v1.1 cycle sequencing kit (Applied Biosystems) and purified by ethanol precipitation. Sequence chromatograms from both strands were obtained on automated sequence analyzer ABI3730XL (Applied Biosystems). For sequence analysis, contig assembly was performed using the software BioNumerics version 5.1 (Applied-Maths, Sint-Martens-Latem, Belgium). Sequence alignments and computation of substitution tables were also performed using the BioNumerics software. For phylogenetic analysis, maximum-likelihood trees were constructed using MEGA version 4 [25] with the Kimura-2 parameter for corrections of multiple substitutions. Reliability of nodes was assessed by boostrap resampling with 1,000 replicates.

### Mice infections

The pathogenicity of the parental P strain, and the selected strains, Z30Alt, Z30B, Z30A, Z30BC or Z30AC, was assayed in 4- to 5-week-old female Swiss mice (OF-1; Charles River, France) by inoculating 10^4^ PFU intraperitoneally into each mouse. The control was inoculated with DMEM supplemented with 10% FBS. Mice surviving at the end of the observation period were bled and their sera tested for IgG by enzyme-linked immunosorbent assay (ELISA) [26]. Two experiments were carried out. In the first, each batch of five mice was infected with a different virus strain: P, Z30Alt, Z30B, Z30A, Z30BC or Z30AC. One batch was used as the control. Mice were kept under observation for 21 days post-inoculation or until death occurred. In the second experiment, we aimed to detect the IgG protective capacity induced by selected clones in inoculated mice. Batches of 12 mice were inoculated with the clones Z30BC and Z30AC, and one batch was used as control. At day 14 post-inoculation, one half of surviving mice from each batch was challenged with 10^4^ PFU of the parental P strain and the other half was inoculated with DMEM. Mice were then observed for 21 days after challenge.

## Results

**Figure 1:**
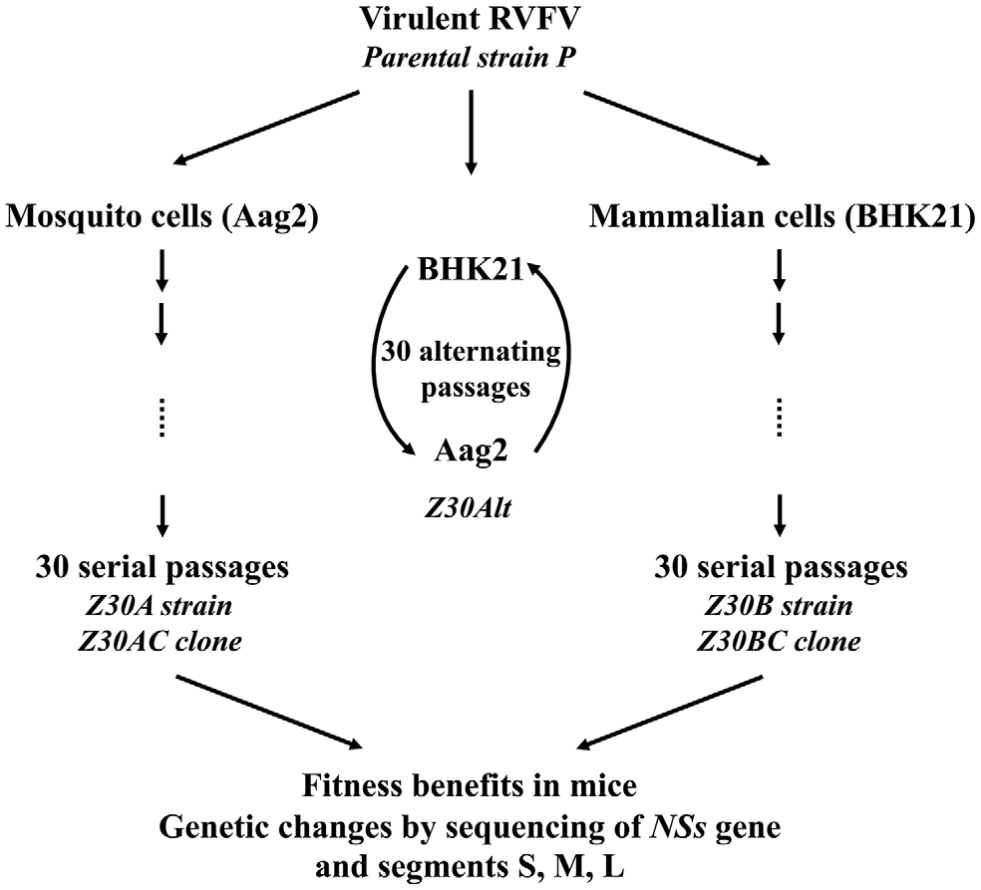
Experimental strategy to test RVFV evolution *in vitro*. The parental P strain virus was subjected to 30 serial passages in BHK21 cells or Aag2 cells, or 30 passages that alternated between BHK21 and Aag2 cells, using a multiplicity of infection (MOI) of 0.1 PFU/cell. The fitness of P (the parental strain), Z30Alt (bulk harvest at the 30^th^ alternating passage in BHK21 and Aag2 cells), Z30B (bulk harvest at the 30^th^ serial passage in BHK21 cells), Z30A (bulk harvest at the 30^th^ serial passage in Aag2 cells), Z30BC (a clone isolated after the 30^th^ serial passage in BHK21 cells) and Z30AC (a clone isolated after the 30^th^ serial passage in Aag2 cells) were tested by inoculation into mice. Genetic changes were detected by sequencing the *NSs* gene carrying the virulence factor.

Figure 1 describes the strategy adopted to test the hypothesis that an alternating host cycle constrains the evolution of RVFV.

### Adaptation to a single cell type results in higher replication rates in the same cell type

**Figure 2:**
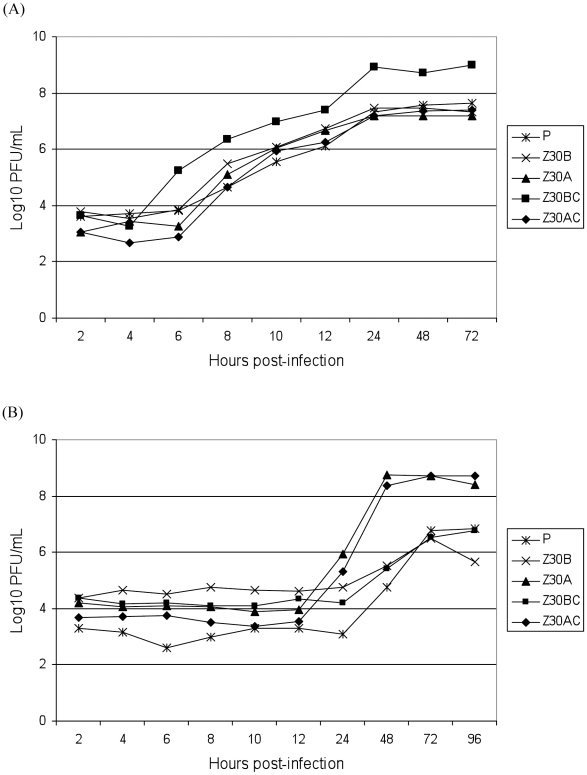
Replication of RVFV strains *in vitro*. BHK21 cells (A) and Aag2 cells (B) were infected at a MOI of 0.1 PFU/cell. After 1 hr of absorption, the inoculum was removed and supernatants were harvested and titrated by serial 10-fold dilutions on Vero cells. The viral strains tested were: P (the parental strain), Z30B (from the 30^th^ serial passage in BHK21 cells), Z30A (from the 30^th^ serial passage in Aag2 cells), Z30BC (a clone selected from the 30^th^ serial passage in BHK21 cells) and Z30AC (a clone selected from the 30^th^ serial passage in Aag2 cells).

To compare the replication of selected RVFV strains (Z30B, Z30A, Z30BC, Z30AC) to the parental P strain, replication kinetics were determined in BHK21 cells and in Aag2 cells. When examining replication rates in BHK21 cells (Figure 2A), all strains gave similar patterns of viral growth, with an initial exponential growth phase until 24 hr post-infection (p.i.) followed by a plateau. However, the Z30BC clone exhibited higher titers than other virus strains, almost 1 log_10_ PFU/mL higher from 6 hr p.i. until 72 hr p.i. Furthermore, viral replication was detectable two hours earlier for Z30BC than for the other four strains tested. This strain reached a maximum titer of around 9 log_10_ PFU/mL from 24 hr p.i. onwards. These results suggest that the Z30BC strain was better adapted to BHK21 cells than other strains were, and that the 30^th^ passage in BHK21 cells (Z30B) from which Z30BC was derived encompassed a mixture of different viral clones with variable capacities to replicate in BHK21 cells. When replication rates in Aag2 cells were analyzed (Figure 2B), differences in titers were clear-cut from 24 hr p.i. onwards. Effectively, Z30A and the Z30AC clone derived from this strain replicated to higher titers than the other three strains tested: ∼4 log_10_ PFU/mL higher at 48 hr p.i. and ∼2 log_10_ PFU/mL higher at 96 hr p.i. Moreover, replication of Z30A and Z30AC was detectable 24 hr earlier than that of the other strains, suggesting their better adaptation to Aag2 cells. In summary, the two selected clones that resulted from serial passages in either BHK21 or Aag2 cells exhibited an increased replication capacity in the corresponding cell type.

### Viral strains isolated after passaging in a single cell type induce attenuation in mice

**Figure 3:**
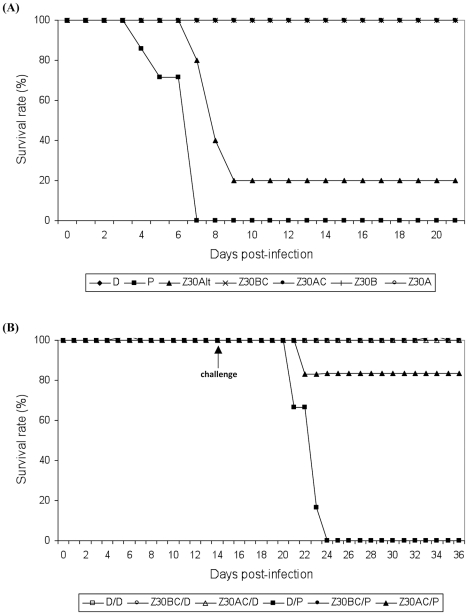
Survival of mice inoculated with RVFV strains. Mice were observed for 21 days post-inoculation (A) or 36 days after challenge with the parental P strain given 14 days post-inoculation (B). The first experiment used batches of mice inoculated with 10^4^ PFU of a viral strain and kept for 21 days post-inoculation. The second experiment used batches of mice inoculated with 10^4^ PFU of Z30BC or Z30AC. At day 14 post-inoculation, one half of surviving mice were challenged with 10^4^ PFU of P and the other half were inoculated with medium. Mice were then observed for 21 days after challenge. The control was inoculated with medium before being challenged by 10^4^ PFU of P. The viral strains tested were: P (the parental strain), Z30Alt (from the 30^th^ alternating passage in BHK21 and Aag2 cells), Z30B (from the 30^th^ serial passage in BHK21 cells), Z30A (from the 30^th^ serial passage in Aag2 cells), Z30BC (a clone selected from the 30^th^ serial passage in BHK21 cells) and Z30AC (a clone selected from the 30^th^ serial passage in Aag2 cells).

We inoculated four-week-old mice intraperitoneally with 10^4^ PFU of one of six viral strains to evaluate their virulence. Strains tested were: P (the parental strain), Z30Alt (isolated at the 30^th^ alternating passage in BHK21 and Aag2 cells), Z30B (pool harvest at the 30^th^ serial passage in BHK21 cells), Z30A (pool harvest at the 30^th^ serial passage in Aag2 cells), Z30BC (clone selected at the 30^th^ serial passage in BHK21 cells) and Z30AC (clone selected at the 30^th^ serial passage in Aag2 cells). Mouse survival rates were recorded over 21 days post-inoculation (Figure 3A). The control, inoculated with Dulbecco's MEM (DMEM) medium, survived 21 days. All batches of mice inoculated with a given viral strain also survived, except for those inoculated with the parental P strain and the 30^th^ alternating passage strain (Figure 3A). All mice inoculated with P died before day 7 post-inoculation, and 4 of 5 treated with Z30Alt died before day 9 post-inoculation. Thus, the 30^th^ alternating passage strain behaved roughly like the parental P strain after 30 passages. All surviving mice were tested for the presence of IgG against RVFV at day 21 post-inoculation, and showed positive compared to the IgG level in non-infected mice (Table S2).

To test the protective effect of infection by clones Z30BC or Z30AC against infection pool harvest with the parental P strain, further batches of mice were first inoculated with the Z30BC or Z30AC clones and then challenged 14 days later by inoculation with the parental P strain. Mortality rates were scored up to 21 days after challenge (Figure 3B). Before challenge, all mice had survived. The control mice started to die five days after challenge, and no controls survived beyond 9 days after challenge. Batches of mice that received one of the two clones at day 0 survived for 36 days. When mice previously inoculated with the Z30BC clone were challenged with the P strain, all mice survived 21 days after challenge. This suggests protection by the Z30BC clone selected in BHK21 cells. Mice sera were all IgG positive (Table S3). In batches of mice first inoculated with the clone Z30AC, one among 6 mice died 8 days after challenge with the parental P strain. The five others survived 21 days after challenge. The sera of the surviving mice were IgG positive (Table S3), suggesting that primary inoculation with the Z30AC clone selected in Aag2 cells could protect mice against a secondary inoculation with the parental P strain.

### *NSs* undergoes large deletions during passages through a single cell type

**Figure 4:**
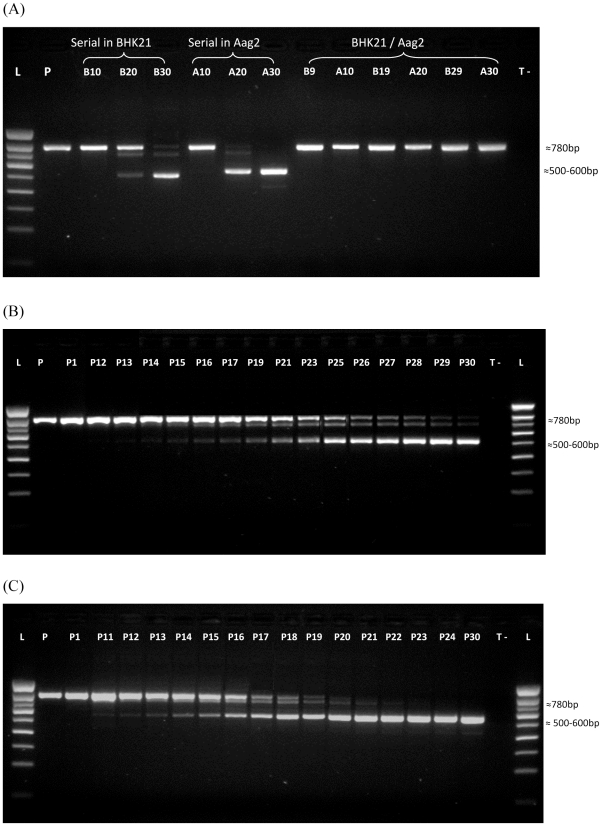
Nature of the *NSs* gene after 10, 20 and 30 passages. (A) Agarose gel electrophoresis showing the *NSs* gene amplified by RT-PCR. Details of serial passages on BHK21 cells (B) and Aag2 cells (C) are given. Total RNAs were extracted from supernatants and RT-PCR targeting the *NSs* gene was conducted. **L**: 100-pb ladder; **P**: parental viral strain; **T-**: negative control.

As the phosphoprotein *NSs* has been shown to be responsible for virulence, we used RT-PCR amplification to monitor the *NSs* gene upon serial or alternating passages through BHK21 and Aag2 cells. Amplification of the parental P strain *NSs* gene generated an amplicon of ∼780 bp (Figure 4A). Upon serial passage in BHK21 cells, PCR products from the 10^th^, 20^th^ and 30^th^ passage exhibited different sized amplicons, with a predominant band at 700–800 bp at the 10^th^ and 20^th^ passages and a band at 500–600 bp at the 30^th^ passage (Figure 4A). When examining the RT-PCR profiles for different serial passages in BHK21 cells in detail, it could be seen that the 500–600 bp band was detectable from the 15^th^ passage, reaching maximum expression from the 25^th^ passage onwards, concomitant with a decrease in the expression of 700–800 bp bands (Figure 4B). The shortening of the *NSs* gene coincided with the loss of cellular lysis in BHK21 cells, evident by the 30^th^ passage. Upon serial passages in Aag2 cells, a band of the expected size corresponding to the *NSs* gene was found at the 10^th^ passage, whereas a smaller band of 500–600 bp was detected at the 20^th^ and 30^th^ passage (Figure 4A). This smaller band was actually found as early as the 11^th^ passage, and became predominant by the 21^st^ passage (Figure 4C), since the 700–800 bp band decreased in quantity from the 20^th^ passage. In contrast, when virus was subjected to alternating passages, only a major band at 700–800 bp was found, irrespective of passage number (Figure 4A).

**Figure 5:**
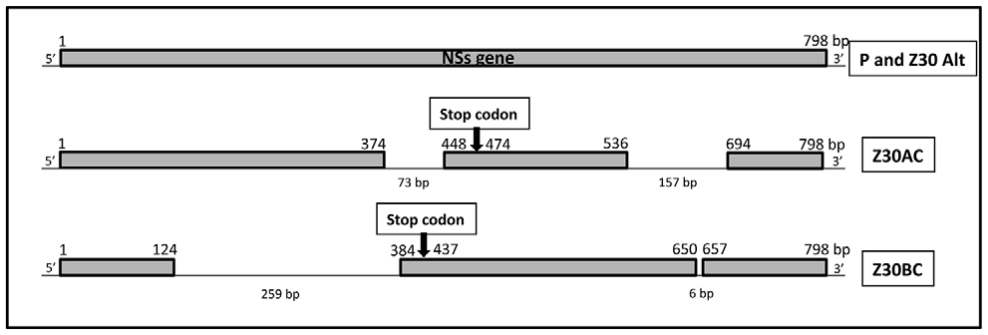
Cartography of the *NSs* gene from the parental P strain and the *in vitro* selected strains, Z30Alt, Z30BC and Z30AC. Z30BC and Z30AC were cloned by plaque purification and amplification on Vero cells. Briefly, cells were incubated for five days under an overlay consisting of DMEM, and the lytic plaques formed were removed individually and dissolved for RNA extraction, RT-PCR and sequencing analysis.

To characterize more precisely the molecular events associated with the emergence of viral variants, clones were analyzed by RT-PCR and sequencing of the *NSs* gene. Virus from the 30^th^ passage in BHK21 or Aag2 cells showed large deletions in *NSs*, while viruses passaged alternately through the two cell types showed no nucleotide changes, suggesting the maintenance of *NSs* integrity during alternating cell-type passages. 48 clones isolated from the 30^th^ serial passage in BHK21 presented two deletions: (i) a deletion of 259 nucleotides (nt) at position 124 that leads to a shift in the *NSs* open reading frame (ORF), introducing a stop codon at position 437, and (ii) a smaller deletion of 6 nt at position 650. Thus, the Z30BC clone has a 533 nt *NSs* gene. 48 clones isolated after 30 passages in Aag2 cells presented two deletions in the *NSs* gene: (i) a deletion of 73 nt at position 374, inducing a shift in the ORF with the introduction of a stop codon at position 474, and (ii) a deletion of 157 nt at position 536. The Z30AC clone thus presents a 568 nt *NSs* gene. Figure 5 summarizes the cartography of the deletions found in the *NSs* gene of clones Z30BC and Z30AC compared to the parental P strain. In contrast, Z30Alt which was isolated from the 30^th^ alternating passage in BHK21 and Aag2 cells had no deletion in the *Nss* gene. These results suggest that all clones containing a shorter *NSs* gene than the parent resulted from a single molecular event in both BHK21 and Aag2 cells; in our experiment, all 48 clones examined from the 30^th^ serial passage presented the same deletions. To further explore the molecular features of the parental P strain and the selected strains (Z30Alt, Z30B and Z30A), the complete sequencing of the three segments S, M and L was achieved. Except for the previously described deletions in the *NSs* gene for the strains Z30B and Z30A, no other deletions or mutations were observed in the S segment for the parental P strain, the Z30B and the Z30A strains. Only one silent mutation was found in the *NSs* gene for the Z30Alt strain (Table S4). Concerning the segments M and L, no deletion event was found for the four strains, and the phylogenetic analysis () confirmed a cluster in the genetic lineage A around the reference strain ZH548 originally isolated in 1977 in Egypt [27]. Nevertheless, the segments M and L presented some mutations, for most non silent, in the three selected strains. As expected, the segment M was the most variable with a total of 11 amino-acid substitutions compared to the segment L with only five amino-acid changes (Table S4). To note, the segment M of the three selected strains have retained the previously described five in-frame AUG-methionine start codons and the different amino-acids involved in glycosylation [27].

## Discussion

Results presented in this paper support the hypothesis that an alternating viral cycle comprising of infection of two distinct hosts constrains the evolution of RVFV. The study model used consisted of abolishing the alternate host environment using a cell culture system. Serial passages in a single cell type led to the loss of virulence. Large deletions were observed in the *NSs* gene, non-essential to viral replication. The rapid emergence of *NSs*-deleted variants in the course of serial passages is likely to result from a selective advantage in their replication rates.

After 30 serial passages in either mammalian BHK21 cells defective in IFN-a/b signaling or mosquito Aag2 cells, single-host adapted viruses from mosquito cells (Z30A and Z30AC) replicated better in mosquito cells. These mosquito-cell adapted viruses reached higher titers (2 log_10_ PFU/mL higher) than non-adapted viruses, and their replication was detectable earlier (24 hr earlier). Interestingly, full adaptation to mammalian cells needed more passages as shown in the Figure 4B where the deleted variant was not totally predominant at the 30^th^ serial passage in BHK21 cells. Replication patterns of the Z30B strain in mammalian cells were similar to those of the parental P strain and of viruses that had bypassed this host. However, the Z30BC clone isolated from the 30^th^ serial passage in BHK21 cells presented the highest replication rate in mammalian cells (replication was detectable 2 hr earlier and reached a titer ∼2 log_10_ PFU higher than for other viruses). This result suggested that a higher number of passages would favor adaptation to BHK21 cells which are defective in IFN-a/b signaling. Viral clones from single-cell passages showed a consistent fitness advantage over the parental P strain in the cell type used for their selection: the Z30BC clone exhibited a fitness advantage in BHK21 cells and the Z30AC clone in Aag2 cells. Thus, our clones isolated from single-host adapted passages present fitness gains in their specific host, and no fitness changes in the bypassed host. Similar results have been obtained for members of various arbovirus families using cell culture model systems [28]–[31] or *in vivo* systems [28], [30], [32]–[35].

Surprisingly, when sequencing the *NSs* gene, that encodes the virulence factor responsible for a general inhibition of cellular RNA synthesis and IFN-ß production, we found that single-host adapted viruses presented large deletions in this gene. Virus passaged in mammalian BHK21 cells defective in IFN-a/b signaling had a deletion of 259 nt introducing a stop-codon in the *NSs* gene. The resulting protein was shortened to 60 amino-acids instead of 265. Virus passaged in mosquito Aag2 cells showed a deletion of 73 nt, again with a stop-codon, causing a shortening of the *NSs* protein to 131 amino-acids. These two distinct deletions in the *NSs* gene following 30 serial passages in each cell type suggest that the viral genome may function differently depending on whether replication is in mammalian or mosquito cells. Thus, while serial passages of RVFV in a single cell type selected for a virus with a truncated *NSs* gene specific to that cell type, alternating passages did not allow the emergence of deletions in the *NSs* gene. Indeed, like the parental P strain, the 30^th^ alternating passage virus Z30Alt did not present any major genetic changes in the *NSs* gene. The fact that the *NSs* gene is dispensable in both single host systems suggests that other mutations are involved in the host adaptation process. Thus, the different non-synonymous mutations identified in the segments M and L should be further explored in this context, particularly since they are mostly specialized depending on host. It is likely that these kinds of deletion events take place spontaneously during viral replication and are selected only in the absence of alternation. Further experiments involving independent serial passages would permit to evaluate the frequency at which the phenomenon occurs. Deletions in the *NSs* gene have been described in a naturally attenuated RVFV (Clone 13) purified from a nonfatal human case in the Central African Republic [16]. This strain has a large internal deletion of 549 nt in the *NSs* gene (∼70% of its length). Animals can survive high infectious doses of Clone 13, up to 10^6^ PFU, without developing any symptoms. Furthermore, Clone 13 has been tested as a vaccine candidate in sheep and cattle that can indeed then elicit a protective response against challenge with a virulent RVFV strain. When the clones we obtained after 30 serial passages in a single cell type were inoculated into mice (at 10^4^ PFU), the animals survived for 21 days and developed protective IgG against challenge with a virulent strain of RVFV. Moreover, most mice inoculated with Z30Alt (isolated at the 30^th^ alternating passage) died 2 days later than did those treated with the parental P strain, and one mouse survived virus inoculation. Having said that, it is known that the outcome of infection is mainly determined by a balance between the rate of viral replication and the immune response, which together limit viral spread [36]. This might explain why one of five mice recovered from infection. Nevertheless, the 30^th^ alternating passage virus Z30Alt retained roughly the same level of virulence as the parental P strain owing to the maintenance of *NSs* integrity.

From our results, we have provided new insights as to how biological constraints such as host alternation are necessary to maintain RVFV integrity and virulence. Contrary to most other arboviruses, RVFV could escape from the alternating host replication cycle to evolve more rapidly, like single-host animal RNA viruses. Thus, our single-host adapted RVFV present large deletions in the *NSs* gene associated with a loss of virulence when inoculated into mice, which develop a long-lasting immunity. In contrast to persistent non-cytolytic replication in insects, arboviruses must replicate to high titers in the mammalian host. This increases the probability of transmission during a blood-meal [37], [38]. By its transfer from wild animals (e.g. buffalo [39]) to livestock, RVFV may intensify direct transmission by contacts with infectious tissues or fluids hosting high viral loads. From our results, viruses selected on mammalian cells may favor attenuation via *NSs* alteration, leading to the maintenance of avirulent RVFV strains. Surprisingly, mosquito cell-specific selection also leads to large deletions in the *NSs* gene, with similar phenotypic consequences. In both cases, virulence will only be restored when alternation between both cell types is initiated in conditions that would reconstitute a complete viral genome by reassortments with a virulent RVFV strain. Such *in vitro* studies should be consolidated with studies using *in vivo* systems. Indeed, vertebrates are subjected to acute infections, with clearance of the virus being triggered by the immune defense system, whereas insect vectors sustain persistent viral replication and are the site of such genetic changes as reassortment or recombination upon co-infection [40]. Such rearrangements may restore virulence, upon the acquisition of a complete *NSs* gene in the course of virus replication [41]. Finally, our results suggest that subtle modifications of selective filters can lead to major genetic changes within a viral population.

## Supporting Information

Figure S1 **Replication kinetics of the parental P strain performed at two MOI (0.1 and 0.01).** The parental P strain was used to infect BHK21 cells (A) and Aag2 cells (B) at two MOI (0.1 and 0.01). Supernatants were harvested at different hours post-infection. Titers in PFU/mL were estimated by serial 10-fold dilutions on Vero cells.
(PDF)

Figure S2 **Phylogenetic analysis of complete nucleotide sequence of the segments M and L.** The complete M and L segments of the parental P strain and the selected strains (Z30Alt, Z30B and Z30A) were analyzed using the ML technique with 1,000 replicate bootstrap values (MEGA version 4), with previously described RVFV strains from the different genetic lineages (A to G) [27].
(PDF)

Table S1 **Primers used for RT-PCR and sequencing.**
(PDF)

Table S2 **RVFV-specific antibodies (IgG) detected at day 21 post-inoculation in mice.** Mice were inoculated with 10^4^ PFU of a RVFV strain. At day 21 post-inoculation, blood samples were tested for IgG detected by ELISA. Whole cell lysate from RVFV infected Vero E6 cells or negative control cell lysate from uninfected Vero E6 cells were diluted in PBS and allowed to absorb onto 96 well plates at +4°C overnight. They were used at 1∶1000. Plates were incubated with blood samples diluted at 1∶100 in 2% skim milk and 0.05% tween 20 in 1× PBS at 37°C for 1 hour. Plates were washed 4 times in PBST (1× PBS with 0.05% tween 20) and then incubated with goat anti-mouse (1∶1000) coupled with peroxydase for 1 hour at 37°C. Plates were washed 4 times in PBST prior to the addition of TMB substrate used according to the manufacturer's instructions. Reactions were stopped after 10 min with the addition of 100 µL of phosphoric acid H_3_PO_4_ (1∶8) and read at 450–620 nm. All samples were run in duplicate and averages were used in the analysis. Absolute values obtained from negative control lysates were subtracted from values obtained from the experimental antigen prior to analysis to control for non-specific binding. D, control DMEM; Z30B, the 30^th^ serial passage in BHK21 cells; Z30A, the 30^th^ serial passage in Aag2 cells; Z30BC, a clone selected from the 30^th^ serial passage in BHK21 cells; Z30AC, a clone selected from the 30^th^ serial passage in Aag2 cells; Z30Alt, the 30^th^ alternating passage in BHK21 and Aag2 cells.
(PDF)

Table S3 **RVFV-specific antibodies (IgG) in mice infected with Z30AC or Z30BC and challenged with P strain.** Two batches of 12 mice received a first dose of 10^4^ PFU of Z30AC or Z30BC, and inoculated 14 days later with 10^4^ PFU of the parental P strain for one half and the other half with DMEM. RVFV-specific antibodies (IgG) were then detected by ELISA in blood samples. Whole cell lysate from RVFV infected Vero E6 cells or negative control cell lysate from uninfected Vero E6 cells were diluted in PBS and allowed to absorb onto 96 well plates at +4°C overnight. They were used at 1∶1000. Plates were incubated with blood samples diluted at 1∶100 in 2% skim milk and 0.05% tween 20 in 1× PBS at 37°C for 1 hour. Plates were washed 4 times in PBST (1× PBS with 0.05% tween 20) and then incubated with goat anti-mouse (1∶1000) coupled with peroxydase for 1 hour at 37°C. Plates were washed 4 times in PBST prior to the addition of TMB substrate used according to the manufacturer's instructions. Reactions were stopped after 10 min with the addition of 100 µL of phosphoric acid H_3_PO_4_ (1∶8) and read at 450–620 nm. All samples were run in duplicate and averages were used in the analysis. Absolute values obtained from negative control lysates were subtracted from values obtained from the experimental antigen prior to analysis to control for non-specific binding. D, control DMEM; Z30BC, a clone selected from the 30^th^ serial passage in BHK21 cells; Z30AC, a clone selected from the 30^th^ serial passage in Aag2 cells; P, the parental strain.
(PDF)

Table S4 **Nucleotide and amino-acid changes (in parentheses) observed in the three segments S, M and L.** Changes were recorded in the parental P strain and the selected strains (Z30Alt, Z30B and Z30A) in reference to the strain ZH548.
(PDF)
