# Supplementary figures and images for: Host Alternation Is Necessary to Maintain the Genome Stability of Rift Valley Fever Virus
Source: PLoS Negl Trop Dis. 2011 May 24;5(5):e1156. doi: 10.1371/journal.pntd.0001156 (PMC3101185; doi:10.1371/journal.pntd.0001156)

(A)

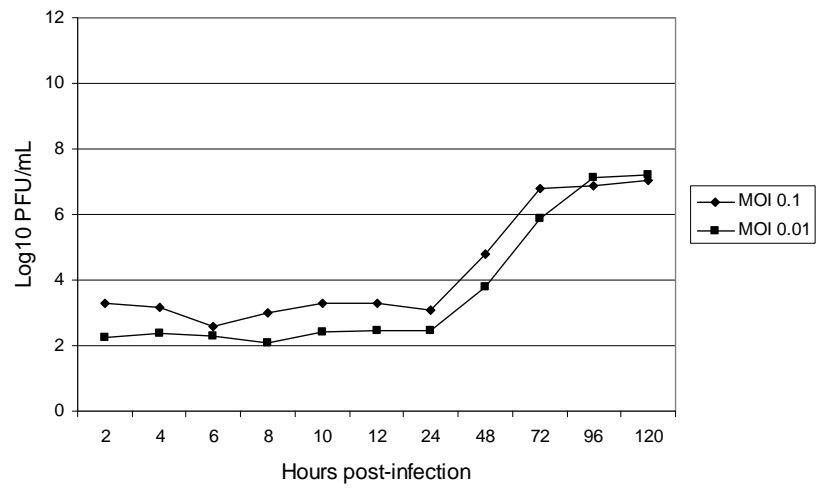

(B)

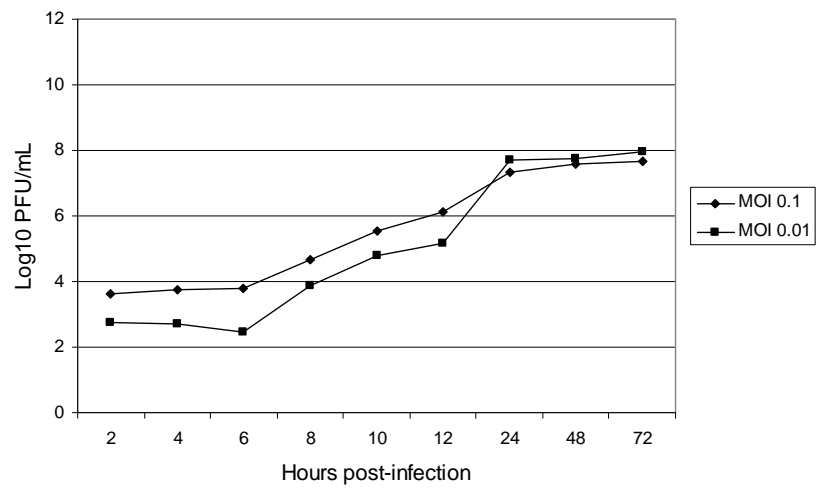

Supplement: Figure S1 — Replication kinetics of the parental P strain performed at two MOI (0.1 and 0.01). The parental P strain was used to infect BHK21 cells (A) and Aag2 cells (B) at two MOI (0.1 and 0.01). Supernatants were harvested at different hours post-infection. Titers in PFU/mL were estimated by serial 10-fold dilutions on Vero cells. (PDF) [file pntd.0001156.s001.pdf]

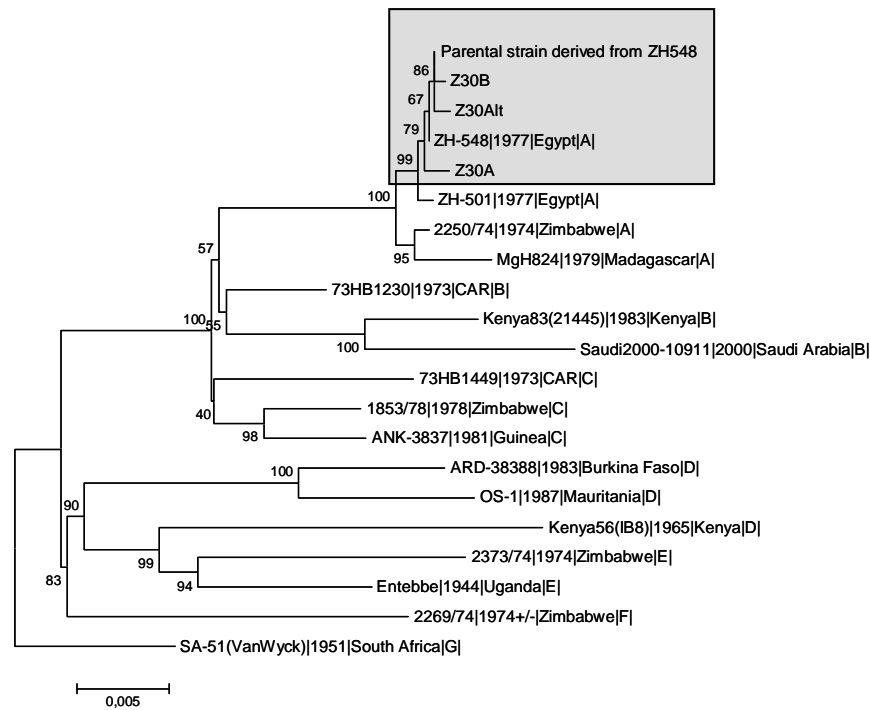

**Segment M**

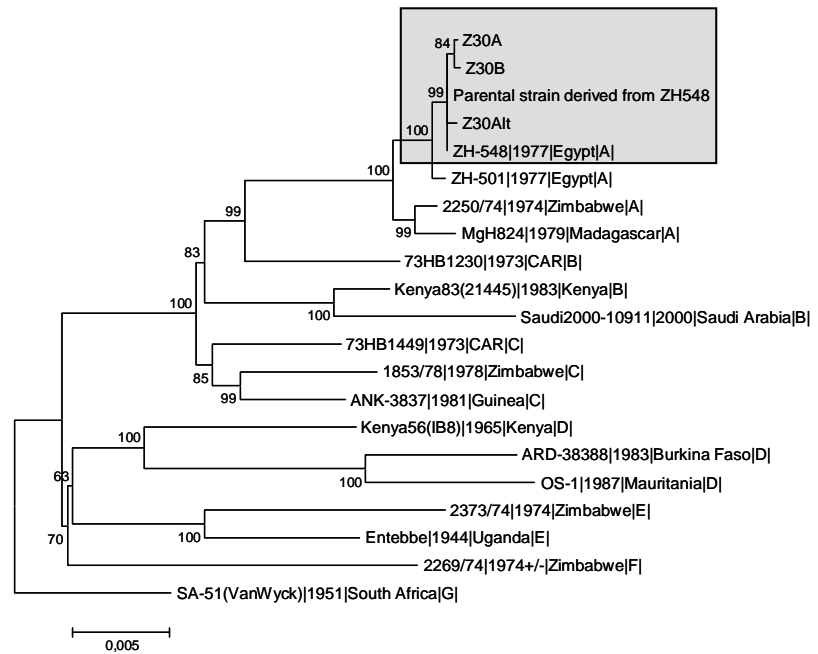

**Segment L**

Supplement: Figure S2 — Phylogenetic analysis of complete nucleotide sequence of the segments M and L. The complete M and L segments of the parental P strain and the selected strains (Z30Alt, Z30B and Z30A) were analyzed using the ML technique with 1,000 replicate bootstrap values (MEGA version 4), with previously described RVFV strains from the different genetic lineages (A to G) [27]. (PDF) [file pntd.0001156.s002.pdf]
