# Supplementary material for: Host Alternation Is Necessary to Maintain the Genome Stability of Rift Valley Fever Virus
Source: PLoS Negl Trop Dis. 2011 May 24;5(5):e1156. doi: 10.1371/journal.pntd.0001156 (PMC3101185; doi:10.1371/journal.pntd.0001156)
Supplement: Table S1 — Primers used for RT-PCR and sequencing. (PDF) [file pntd.0001156.s003.pdf]

Fragment	Primer (a)	Sequence (5' to 3')
S1	pf_RiftS_0F	ACACAAAGACCCCCTAGTGC
	pf_RiftS_700R	GCTCTCCTCCATAAGAAC
S2	Rift-S-NS2g	TGATTTGCAGAGTGGTCGTC
	Rift-S-NScag	CCTTAACCTCTAATCAAC
S3	pf_RiftS_560F	ACCTGTGCCTGTTGCCAGGC
	pf_RiftS_1260R	TACCATGGACGGCCTATCC
S4	Rift-S-int771F	CAGATGTTGAGATGGAATCAG
	Rift_S-int1130R	GCTCACTCTCTGTATCTGCTGC
S5	pf_RiftS_1050F	TTCATTGGCTGCGTGAACG
	pf_RiftS_1690R	ACACAAAGCTCCCTAGAG
M1	pf_RiftM_0F	ACACAAAGATGGTGCATT
	MRV5g_RiftM_758R	GGACAGTGTGTCTTC
M2	pf_RiftM_560F	CCTGTGACATATGCTGGG
	MRV3g_RiftM_1326R	CCTGACCCATTAGCATG
M3	pf_RiftM_1190F	AAGAGGGAGCTCAAAAG
	pf_RiftM_2100R	GTTCTGAACATGCTGATGC
M4	pf_RiftM_1750F	CCAGTGTCACACTGCTCTC
	pf_RiftM_2450R	TCGCATGGTCGTGCTTTCC
M5	pf_RiftM_2310F	CTGGGTCCTTTAGCCCTA
	pf_RiftM_3010R	CTATTTCCTTTTGAAGCTGC
M6	pf_RiftM_2870F	CCAAACCTTATCTCATAC
	pf_RiftM_3570R	CTGTTCCTCCAAGATATATAAG
M7	pf_RiftM_3360F	TGATAGCCATTGATCCAT
	pf_RiftM_3885R	ACACAAAGACCGGTGCAAC
L1	pf_RiftL_0F	ACACAAAGGCGCCCAATCATG
	pf_RiftL_700R	AGGACACCATGGCTGGCAAC
L2	pf_RiftL_500F	GATCTCAGGGCAGGACTGTTG
	pf_RiftL_1280R	CCATTAACTCCTAAAGCAGC
L3	pf_RiftL_1120F	ACCTAGAGGAGATTGAGAG
	pf_RiftL_1820R	TTGCTTAGCTTATAAGAAAC
L4	pf_RiftL_1680F	CCACTTCCAGCAAAAGCC
	pf_RiftL_2380R	GTCTAAGGTGCTCAAGTTC
L5	pf_RiftL_2240F	TTCAGGGCGTCCACTGCTTG
	pf_RiftL_2940R	CCTGATGCCATGTGTTTCAGG
L6	pf_RiftL_2800F	TCTATGTGATGGGTGCAGAGG
	pf_RiftL_3500R	CGGAAGCATATAGCTGCG
L7	pf_RiftL_3360F	AAGGTTGCTCCTGAGATGAG
	pf_RiftL_4060R	TTAGAGCGCCACCTGGGCTC

L8	pf_RiftL_3920F	CAAAGCTATCACCAGAACTG
	pf_RiftL_4620R	AAACCACTTGTCTGACACC
L9	pf_RiftL_4480F	CTATAGTTTACAACAAGGG
	pf_RiftL_5180R	ACTTTAGATTGTTGTCTC
L10	pf_RiftL_5040F	AAGGAATCAGATGGTAAGAC
	pf_RiftL_5740R	TAAATTTTGGGGCTACAGAC
L11	pf_RiftL_5600F	TTACACTGCATCAGACAATG
	pf_RiftL_6300R	AATCTCTGAGAGTCTGGTTC
L12	pf_RiftL_6160F	CAATTATTTGAGAGGGAGC
	pf_RiftL_6606R	ACACAAAGGCGCCCAATC

(a) The primer name indicates their position and direction in the nucleotide sequence of the ZH548 strain genome
