# Supplementary material for: Host Alternation Is Necessary to Maintain the Genome Stability of Rift Valley Fever Virus
Source: PLoS Negl Trop Dis. 2011 May 24;5(5):e1156. doi: 10.1371/journal.pntd.0001156 (PMC3101185; doi:10.1371/journal.pntd.0001156)
Supplement: Table S2 — RVFV-specific antibodies (IgG) detected at day 21 post-inoculation in mice. Mice were inoculated with 104 PFU of a RVFV strain. At day 21 post-inoculation, blood samples were tested for IgG detected by ELISA. Whole cell lysate from RVFV infected Vero E6 cells or negative control cell lysate from uninfected Vero E6 cells were diluted in PBS and allowed to absorb onto 96 well plates at +4°C overnight. They were used at 1∶1000. Plates were incubated with blood samples diluted at 1∶100 in 2% skim milk and 0.05% tween 20 in 1× PBS at 37°C for 1 hour. Plates were washed 4 times in PBST (1× PBS with 0.05% tween 20) and then incubated with goat anti-mouse (1∶1000) coupled with peroxydase for 1 hour at 37°C. Plates were washed 4 times in PBST prior to the addition of TMB substrate used according to the manufacturer's instructions. Reactions were stopped after 10 min with the addition of 100 µL of phosphoric acid H3PO4 (1∶8) and read at 450–620 nm. All samples were run in duplicate and averages were used in the analysis. Absolute values obtained from negative control lysates were subtracted from values obtained from the experimental antigen prior to analysis to control for non-specific binding. D, control DMEM; Z30B, the 30th serial passage in BHK21 cells; Z30A, the 30th serial passage in Aag2 cells; Z30BC, a clone selected from the 30th serial passage in BHK21 cells; Z30AC, a clone selected from the 30th serial passage in Aag2 cells; Z30Alt, the 30th alternating passage in BHK21 and Aag2 cells. (PDF) [file pntd.0001156.s004.pdf]

	N°	Replicate 1	Replicate 2
D	1	0.05	0.06
	2	0.05	0.04
	3	0.04	0.07
	4	0.04	0.04
	5	0.07	0.07
Z30B	1	3.60	3.61
	2	3.48	3.58
	3	0.06	0.14
	4	3.48	3.50
	5	3.55	3.70
Z30A	1	3.34	3.23
	2	3.48	3.64
	3	3.56	3.7
	4	3.55	3.68
	5	3.50	3.67
Z30BC	1	2.30	2.90
	2	3.14	3.09
	3	3.33	3.09
	4	3.60	3.52
	5	3.45	3.32
Z30AC	1	3.36	3.42
	2	3.30	3.48
	3	3.13	3.20
	4	3.60	3.49
	5	3.33	3.05
Z30Alt	5	2.23	2.31
