# Supplementary material for: Host Alternation Is Necessary to Maintain the Genome Stability of Rift Valley Fever Virus
Source: PLoS Negl Trop Dis. 2011 May 24;5(5):e1156. doi: 10.1371/journal.pntd.0001156 (PMC3101185; doi:10.1371/journal.pntd.0001156)
Supplement: Table S3 — RVFV-specific antibodies (IgG) in mice infected with Z30AC or Z30BC and challenged with P strain. Two batches of 12 mice received a first dose of 104 PFU of Z30AC or Z30BC, and inoculated 14 days later with 104 PFU of the parental P strain for one half and the other half with DMEM. RVFV-specific antibodies (IgG) were then detected by ELISA in blood samples. Whole cell lysate from RVFV infected Vero E6 cells or negative control cell lysate from uninfected Vero E6 cells were diluted in PBS and allowed to absorb onto 96 well plates at +4°C overnight. They were used at 1∶1000. Plates were incubated with blood samples diluted at 1∶100 in 2% skim milk and 0.05% tween 20 in 1× PBS at 37°C for 1 hour. Plates were washed 4 times in PBST (1× PBS with 0.05% tween 20) and then incubated with goat anti-mouse (1∶1000) coupled with peroxydase for 1 hour at 37°C. Plates were washed 4 times in PBST prior to the addition of TMB substrate used according to the manufacturer's instructions. Reactions were stopped after 10 min with the addition of 100 µL of phosphoric acid H3PO4 (1∶8) and read at 450–620 nm. All samples were run in duplicate and averages were used in the analysis. Absolute values obtained from negative control lysates were subtracted from values obtained from the experimental antigen prior to analysis to control for non-specific binding. D, control DMEM; Z30BC, a clone selected from the 30th serial passage in BHK21 cells; Z30AC, a clone selected from the 30th serial passage in Aag2 cells; P, the parental strain. (PDF) [file pntd.0001156.s005.pdf]

	N°	Replicate 1	Replicate 2
D / D	1	0.01	0.02
	2	0.02	0.03
	3	0.01	0.01
	4	0.01	0.01
	5	0.02	0.02
	6	0.01	0.01
Z30BC / D	1	0.94	1.10
	2	1.17	1.13
	3	0.60	0.75
	4	0.67	0.80
	5	0.52	0.57
	6	0.74	0.83
Z30AC / D	1	1.04	1.14
	2	0.88	0.94
	3	1.48	1.66
	4	0.99	1.05
	5	0.19	0.20
	6	1.16	1.19
Z30BC / P	1	1.64	1.84
	2	1.38	1.44
	3	0.81	0.83
	4	0.84	0.81
	5	2.10	2.03
	6	0.84	0.99
Z30AC / P	2	1.66	1.51
	3	1.40	1.48
	4	0.78	0.91
	5	0.57	0.72
	6	1.43	1.43
