# Supplementary material for: Host Alternation Is Necessary to Maintain the Genome Stability of Rift Valley Fever Virus
Source: PLoS Negl Trop Dis. 2011 May 24;5(5):e1156. doi: 10.1371/journal.pntd.0001156 (PMC3101185; doi:10.1371/journal.pntd.0001156)
Supplement: Table S4 — Nucleotide and amino-acid changes (in parentheses) observed in the three segments S, M and L. Changes were recorded in the parental P strain and the selected strains (Z30Alt, Z30B and Z30A) in reference to the strain ZH548. (PDF) [file pntd.0001156.s006.pdf]

Segment	S
position on CDS	592
Parental strain	T
Z30A	Deletion zone
Z30B	T
Z30Alt	C
ZH548	T

Segment	M											
position on CDS	188	239	292	448	490	695	1292	2168	2239	2738	3050	3192
Parental strain	T	T	G	G	A	T	A	C	T (I747L)	A	A	T
Z30A	T	C (I80T)	A (D98N)	G	G (K164E)	A (L232Q)	G (N431S)	C	A	G (Y913C)	A	T
Z30B	T	T	G	G	A	T	A	C	T (I747L)	A	G (N1017S)	C
Z30Alt	A (I63K)	T	G	A (V150I)	A	T	A	T (A723V)	T (I747L)	A	A	T
ZH548	T	T	G	G	A	T	A	C	A	A	A	T

Segment	L							
position on CDS	132	1400	3919	4366	4872	4916	5412	6264
Parental strain	T	A	A	A	A	A	A	T
Z30A	T	G (D467G)	A	A	T	G (E1639G)	A	T
Z30B	T	A	A	G (M1456V)	T	G (E1639G)	T	T
Z30Alt	C	A	T(T1307S)	A	A	A	A	A (D2088E)
ZH548	T	A	A	A	A	A	A	T
